# Supplementary material for: DNA N6-methyladenine is dynamically regulated in the mouse brain following environmental stress
Source: Nat Commun. 2017 Oct 24;8:1122. doi: 10.1038/s41467-017-01195-y (PMC5654764; doi:10.1038/s41467-017-01195-y)
Supplement: Supplementary file 1 — Supplementary Information [file 41467_2017_1195_MOESM1_ESM.pdf]

Supplementary Figures

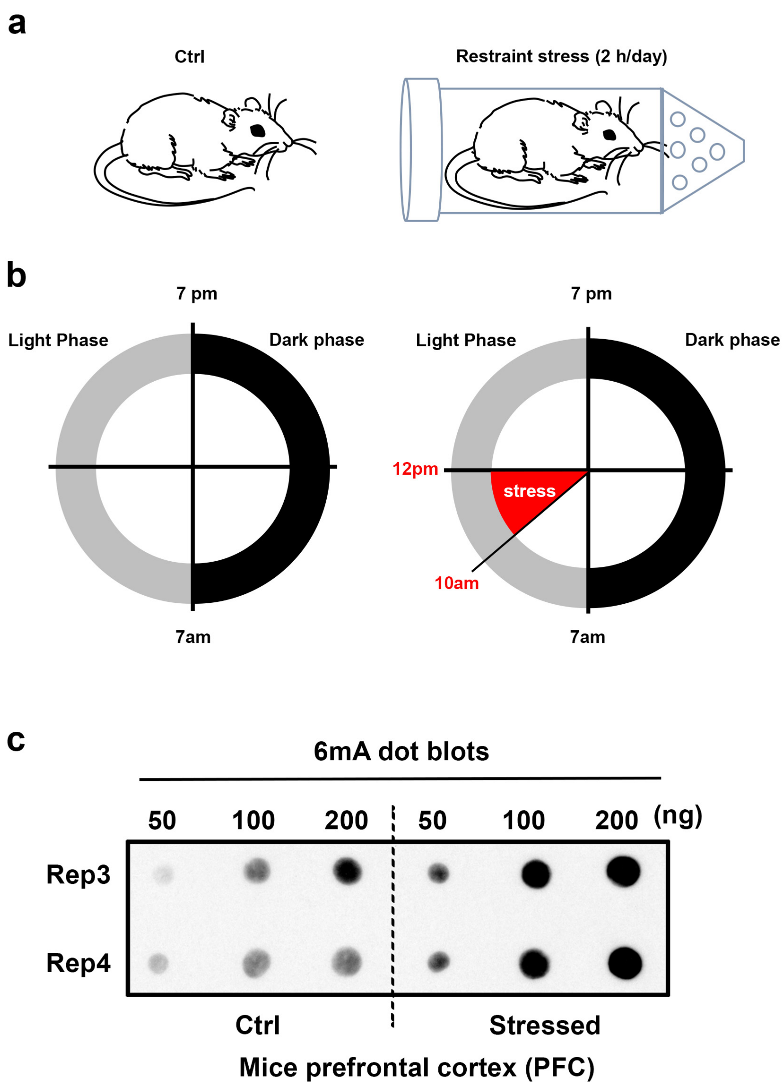

Supplementary Figure 1. Restraint stress increased 6mA level in PFC. (**a**) Mice subjected to stress were individually placed into a customized well-ventilated 50-ml conical tube, in which the mice could change from the supine to prone position but were unable to move forward or backward. The non-stressed littermate controls remained undisturbed in their home cages. (**b**) Timeline for restraint experiments. The experiments were performed daily from 10 am to 12 pm for 2 hours in the original home cages, and the mice were released from the tube afterwards. The total duration of the experiments was 14 days. (**c**) 6mA specific dot blots on two additional replicates from another batch of stress experiments separated from Fig. 1d confirmed the accumulation of 6mA in mice PFC upon stress observed in Fig. 1c.

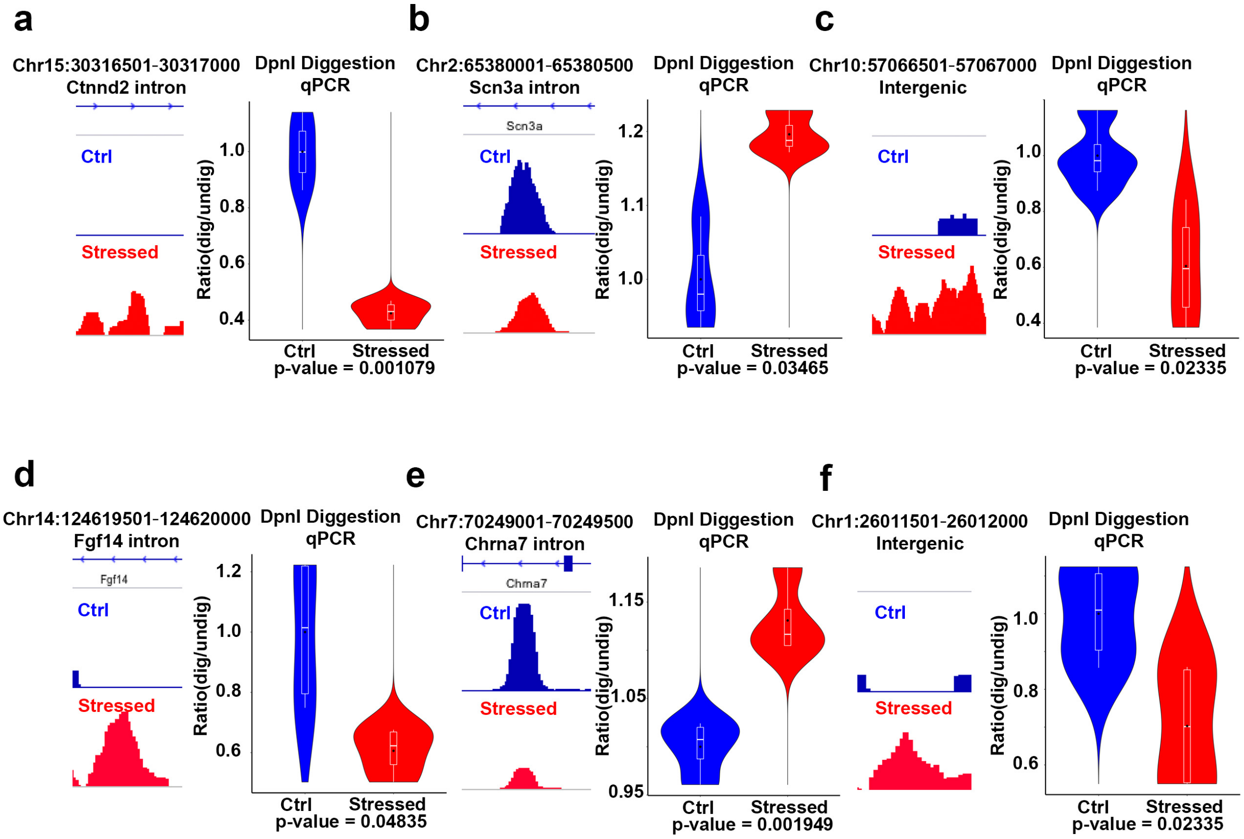

Supplementary Figure 2. Validation of dynamic 6mA regions by 6mA-sensitive restriction enzyme digestion. (**a-f**) 6mA-sensitive restriction enzyme DpnI that preferentially cleaves methylated adenosine at GATC/CATC/GATG sites was used to validate regions identified in Fig. 2a. The percentages of 6mA in either control or stressed PFC were assessed by qPCR amplification of digested DNA using undigested DNA as a control (digested/undigested). Inverse correlation between 6mA reads distribution and qPCR fold changes were observed to confirm the 6mA dynamic changes in these regions. p values were indicated (unpaired *t*-test).

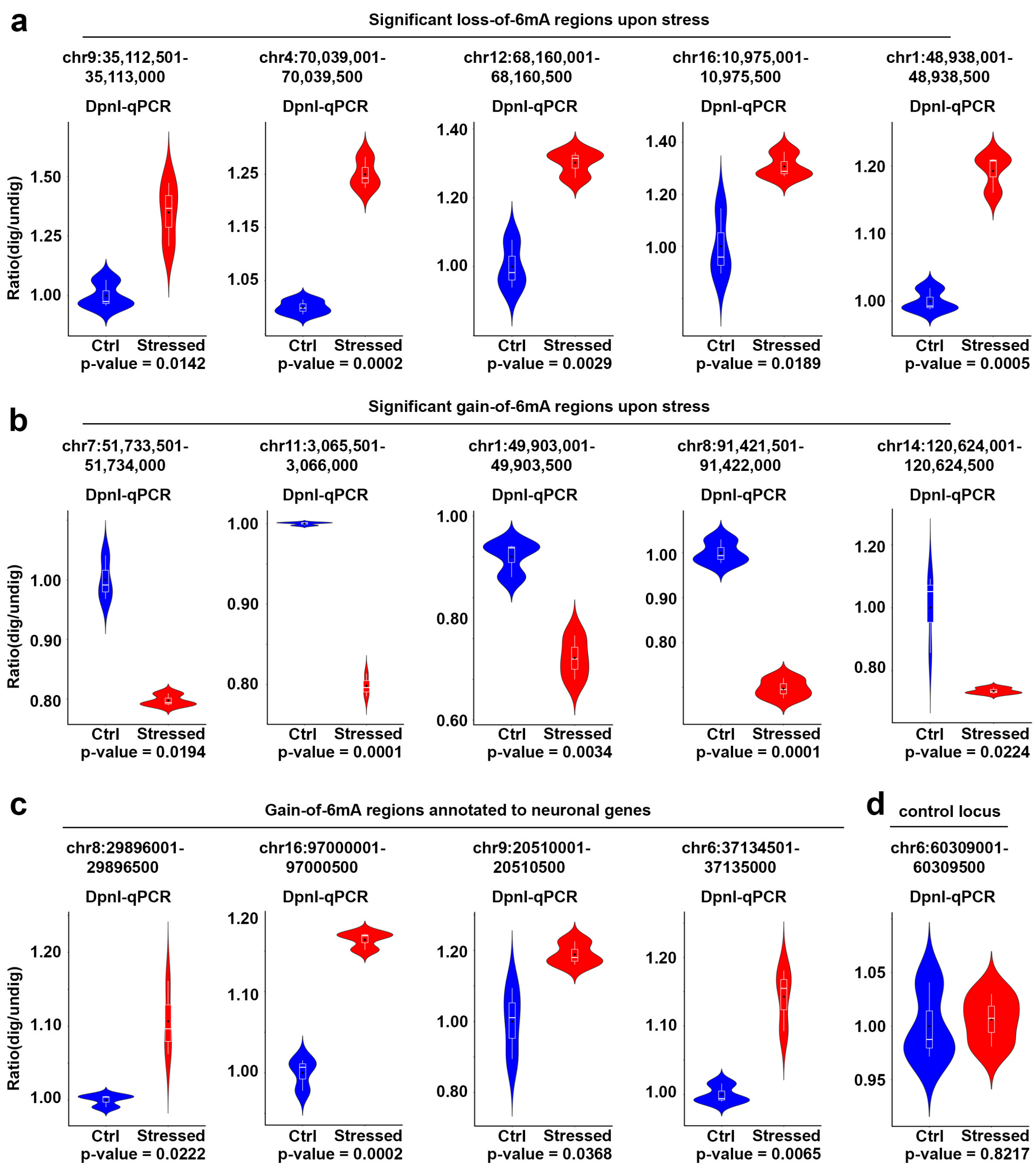

Supplementary Figure 3. Additional validation of dynamic 6mA regions by 6mA-sensitive restriction enzyme digestion. (**a**) DpnI-qPCR was utilized to validate random selected loss-of-6mA regions, p-values are indicated. (**b**) DpnI-qPCR was utilized to validate random selected gain-of-6mA regions, p-values are indicated. (**c**) DpnI-qPCR was utilized to validate random selected loss-of-6mA regions annotated to genes involved in neurodevelopment and neuronal functions, p-values are indicated. (**d**) A control locus that did not show significant 6mA alteration was utilized as a DpnI-qPCR control.

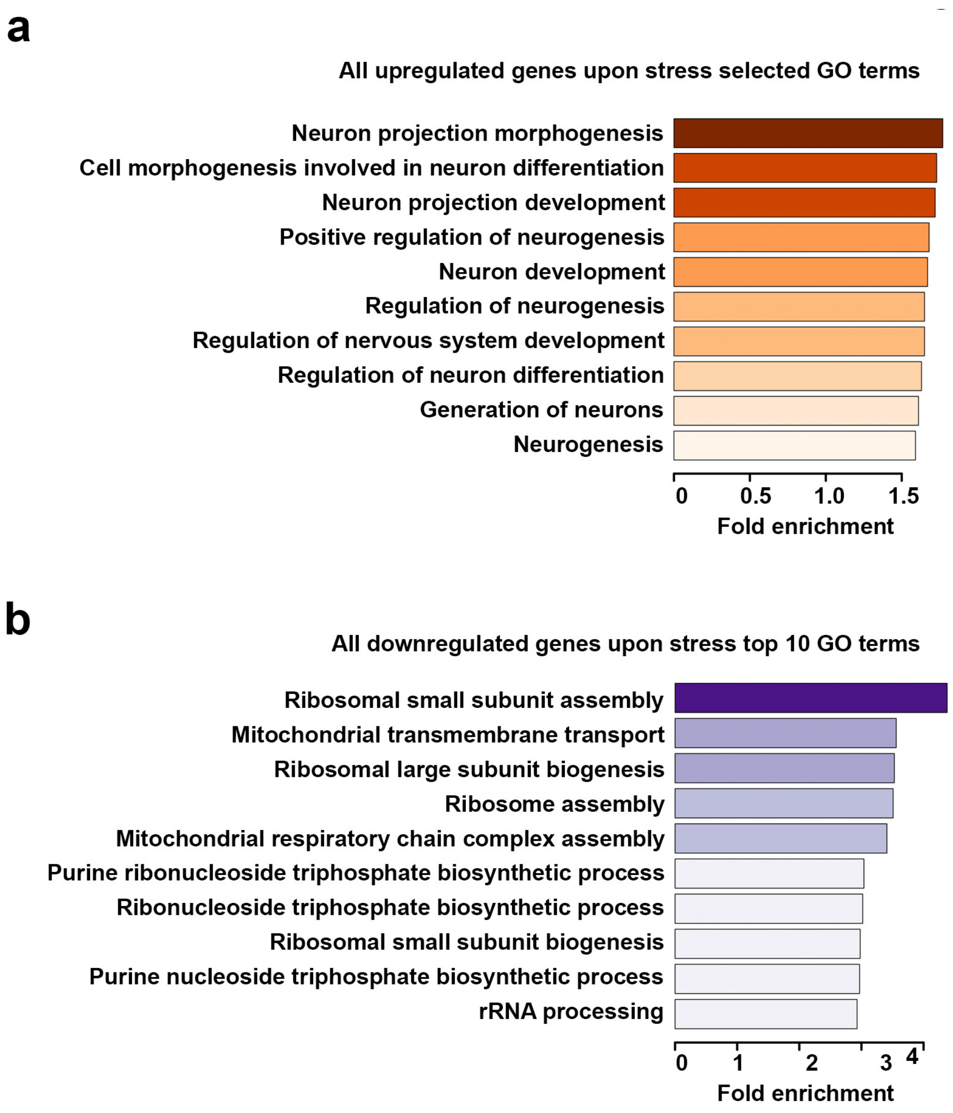

Supplementary Figure 4. Strong enrichment of neuronal function and developmental pathways in upregulated genes upon stress. (**a**) Upregulated genes upon stress were enriched in biological pathways related to neuronal functions. Fold enrichment of each GO term is indicated by x-axis and bar color. Selected neuronal terms in top 50 ranking were shown. (**b**) No biological pathways related to neuronal functions were observed in downregulated genes upon stress. Top 10 GO terms were shown.

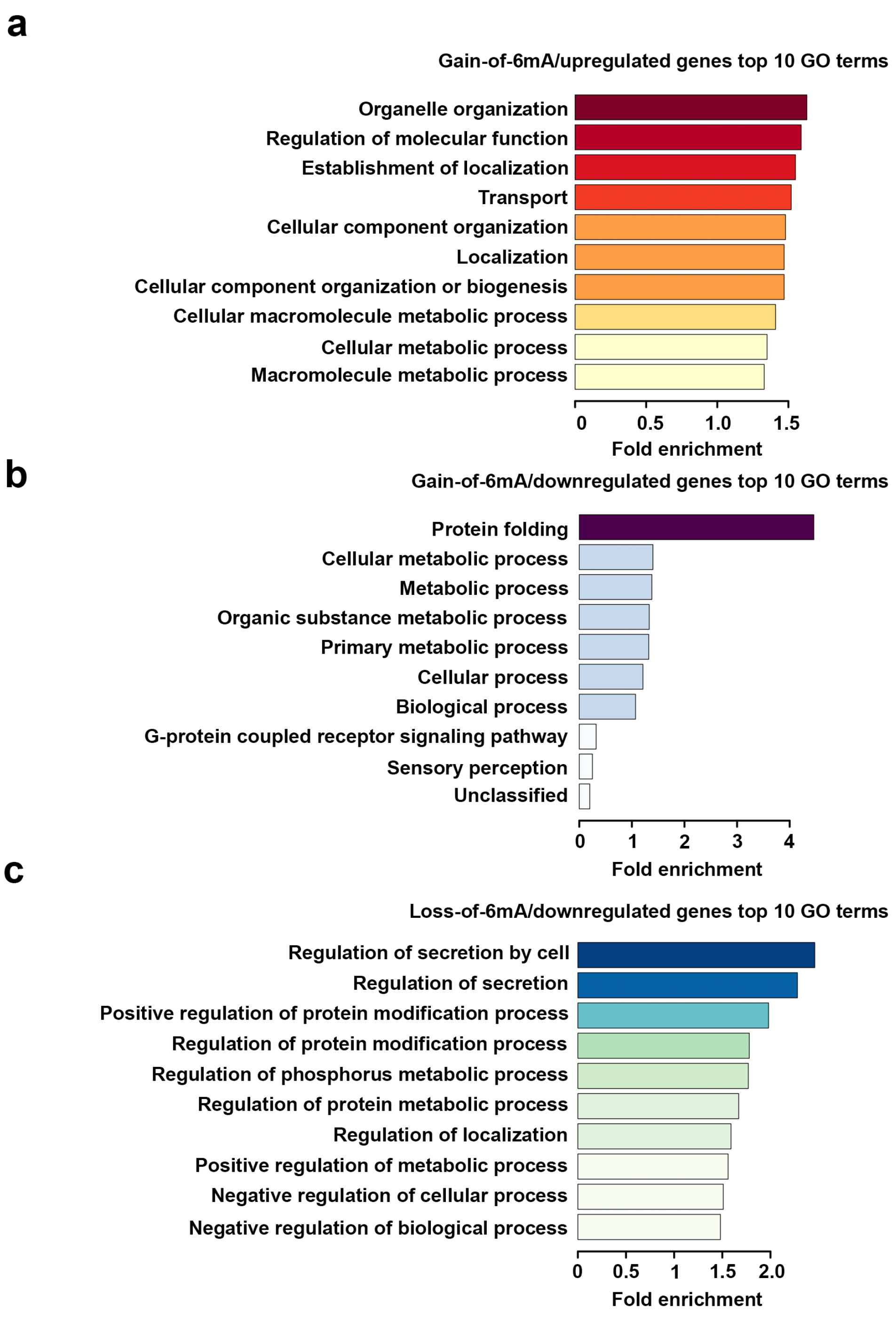

Supplementary Figure 5. Correlation between 6mA dynamics with transcription changes upon stress. (**a**) Gain-of-6mA/upregulated genes, (**b**) Gain-of-6mA/downregulated and (**c**) Loss-of-6mA/downregulated genes were subjected to GO analyses. Top 10 GO terms are shown. Fold enrichment of each GO term is indicated by x-axis and bar color.

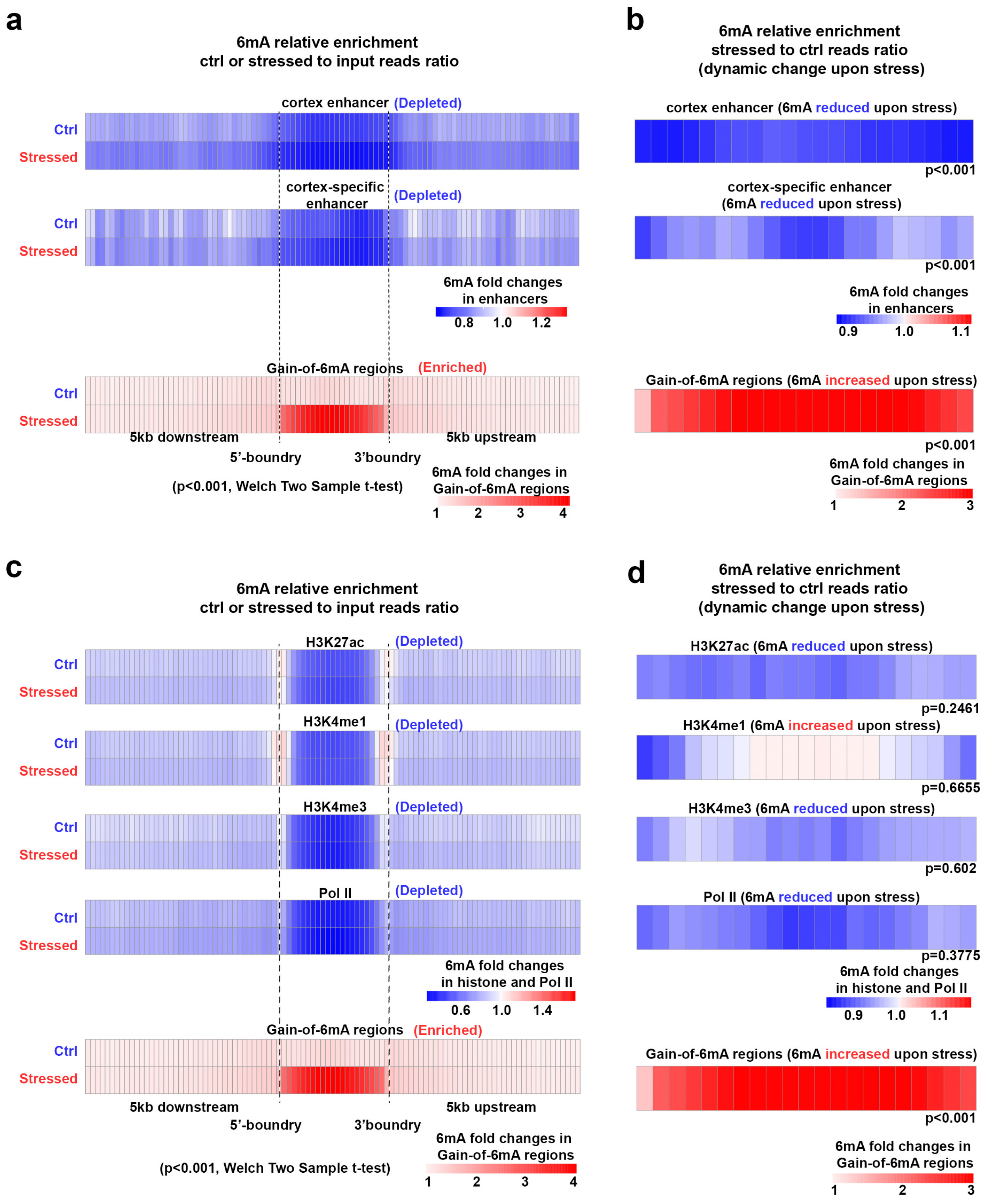

Supplementary Figure 6. 6mA was depleted in cortex enhancers and further loss upon stress. (**a**) Average fold change between 6mA normalized reads (both control and stressed PFC) versus non-enriched input DNA were calculated in cortex general enhancers and cortex-specific enhancers regions plus 5kb upstream and downstream flanking regions. Average fold change

was plotted in Heatmap view. Red plots (fold change > 1) indicate enrichment over input whereas blue plots (fold change < 1) indicate depletion. Gain-of-6mA regions were utilized as positive controls. P-values are indicated (unpaired *t-test*). 6mA was depleted in both cortex general enhancers and cortex-specific enhancers. (**b**) Average fold change ratio between stressed and control 6mA normalized reads were calculated to indicate the 6mA dynamic changes upon stress in cortex general enhancers and cortex-specific enhancers. Average fold change was plotted in Heatmap view. Red plots (fold change > 1) indicate gain-of-6mA in these regions upon stress, whereas blue plots (fold change < 1) indicate loss-of-6mA in these regions upon stress. Gain-of-6mA regions were utilized as positive controls. P-values are indicated (unpaired *t-test*). 6mA was further depleted in both cortex general enhancers and cortex-specific enhancers upon stress (**c**) Average fold change between 6mA normalized reads (both control and stressed PFC) versus non-enriched input DNA were calculated in H3K27ac, H3K4me1 and Pol II regions plus 5kb upstream and downstream flanking regions. 6mA were significantly depleted in these regions versus input. (**d**) Average fold change ratio between stressed and control 6mA normalized reads were calculated to indicate the dynamic 6mA changes upon stress in H3K27ac, H3K4me1 and Pol II regions. No significant 6mA changes were observed in these regions upon stress.

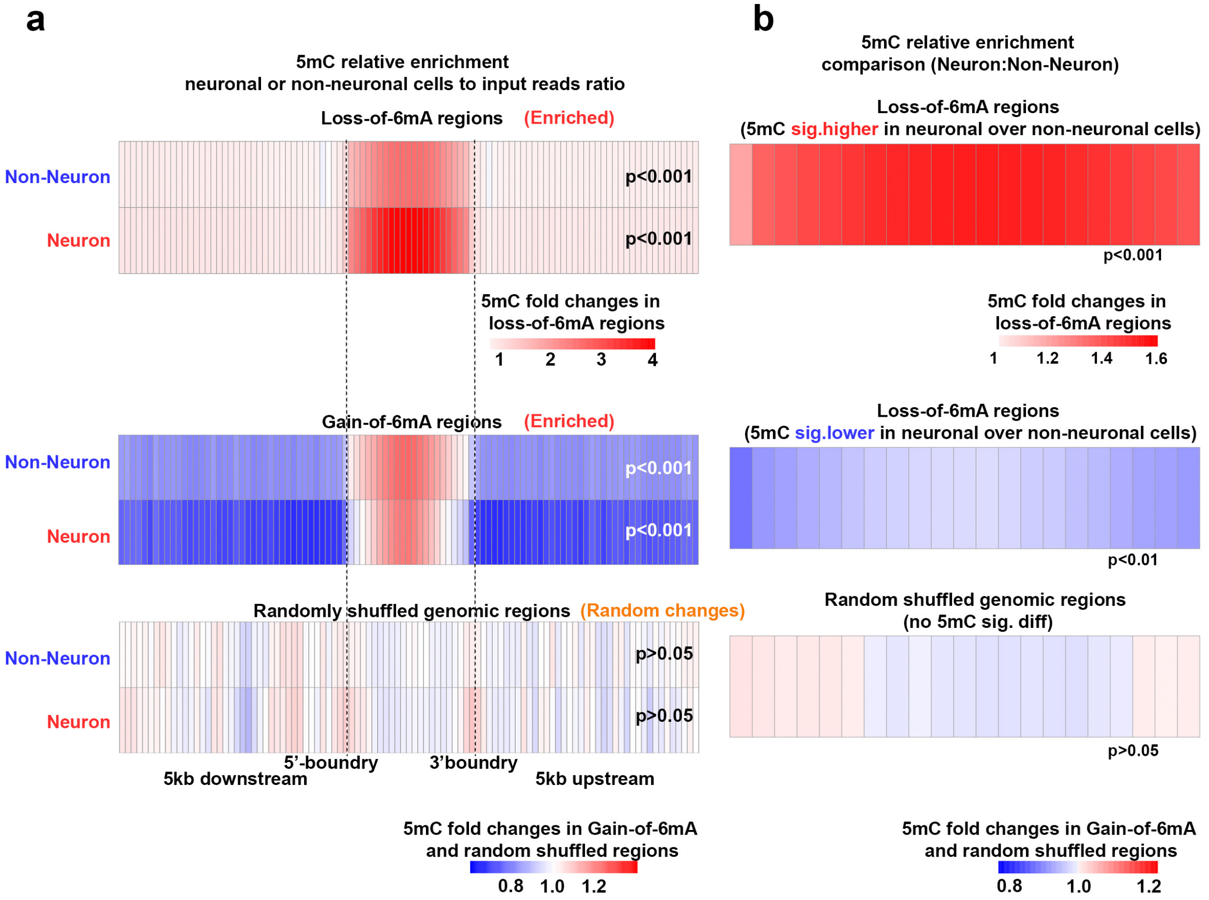

Supplementary Figure 7. 6mA correlated with cytosine methylation, particularly in cortical neurons. (**a**) Average fold change between MeDIP-seq normalized reads (both non-neuronal cells and neuronal cells) versus non-enriched input DNA were calculated in both significant gain- and loss-of-6mA regions plus 5kb upstream and downstream flanking regions. Average fold change was plotted in Heatmap view. Red plots (fold change > 1) indicate enrichment over input whereas blue plots (fold change < 1) indicate depletion. Randomly shuffled loss-of-6mA regions were utilized as negative controls. P-values are indicated (unpaired *t-test*). Methylated cytosines were enriched on regions with higher 6mA in controls. (**b**) Average fold change ratio between neuronal and non-neuronal mC normalized reads were calculated to indicate the mC dynamic changes between neuronal and non-neuronal cells in both significant gain- and loss-of-6mA regions. Average fold change was plotted in Heatmap view. Red plots (fold change > 1) indicate gain-of-6mA in these regions upon stress, whereas blue plots (fold change < 1) indicate loss-of-6mA in these regions upon stress. Randomly shuffled loss-of-6mA regions were utilized as negative controls. P-values are indicated (unpaired *t-test*). Methylated cytosine were particularly enriched in neuronal cells than non-neuronal cells.

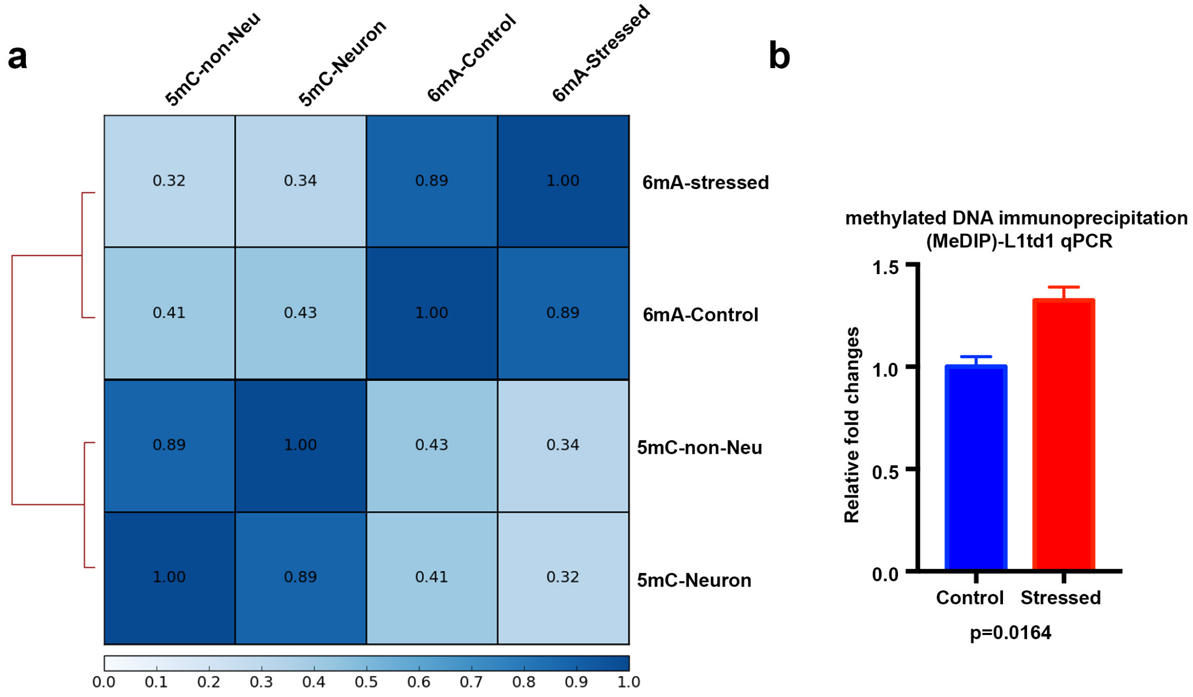

Supplementary Figure 8. Stress-induced DNA methylation changes in LINE transposons. (**a**) Global Spearman correlations between 6mA and 5mC were performed by deepTools. Correlation coefficients and the clusters were plotted. 6mA profile showed highest correlation with 5mC in neuronal cells. (**b**) Methylated DNA immunoprecipitation (MeDIP) were performed from control and stressed PFC DNA. Significant increase of cytosine methylation was detected at the L1td1 locus.

Intragenic loss-of-6mA on upregulated genes	P-value	Predicted binding factor
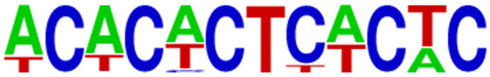	1e-119	Egr2
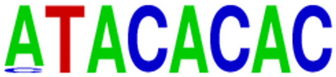	1e-47	Foxh1
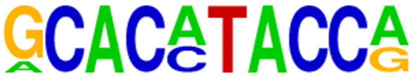	1e-19	Hif2a

Supplementary Figure 9. Motif search on loss-of-6mA regions associated with upregulated genes predicted binding of several transcription factors. HOMER was used to generate common motifs from intragenic loss-of-6mA associated with upregulated genes. Three top-ranked motifs were displayed with their p-values and predicted binding proteins.

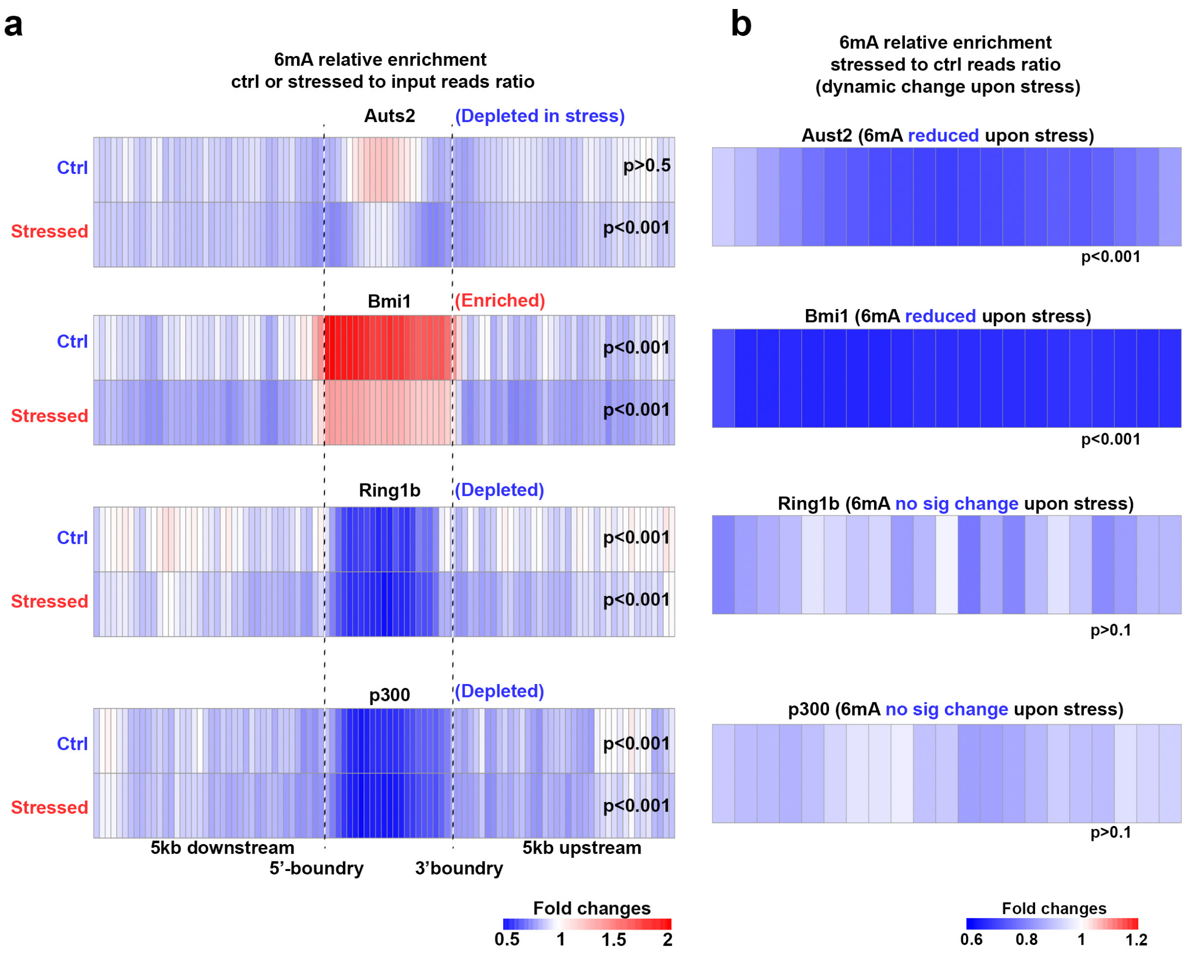

Supplementary Figure 10. 6mA was particularly enriched in Bmi1 binding sites. (**a**) Average fold change between 6mA normalized reads (both control and stressed PFC) versus non-enriched input DNA were calculated in Polycomb protein Aust2, Bmi1, Ring1b and transcription factor p300 binding sites plus 5kb upstream and downstream flanking regions. Average fold change was plotted in Heatmap view. Red plots (fold change > 1) indicate enrichment over input whereas blue plots (fold change < 1) indicate depletion. P-values are indicated (unpaired *t-test*). 6mA was significantly enriched in Bmi1 binding sites but depleted in Ring1b and p300 binding sites. 6mA in control PFC was slightly enriched in Aust2 binding sites and became significantly depleted upon stress (**b**) Average fold change ratio between stressed and control 6mA normalized reads were calculated to indicate the 6mA dynamic changes upon stress in Polycomb protein Aust2, Bmi1, Ring1b and transcription factor p300 binding sites. Average fold change was plotted in Heatmap view. Red plots (fold change > 1) indicate gain-of-6mA in these regions upon stress, whereas blue plots (fold change < 1) indicate loss-of-6mA in these regions upon stress. Gain-of-6mA regions were utilized as positive controls. P-values are indicated (unpaired *t-test*). 6mA were significantly depleted in both Aust2 and Bmi1 binding sites upon stress.

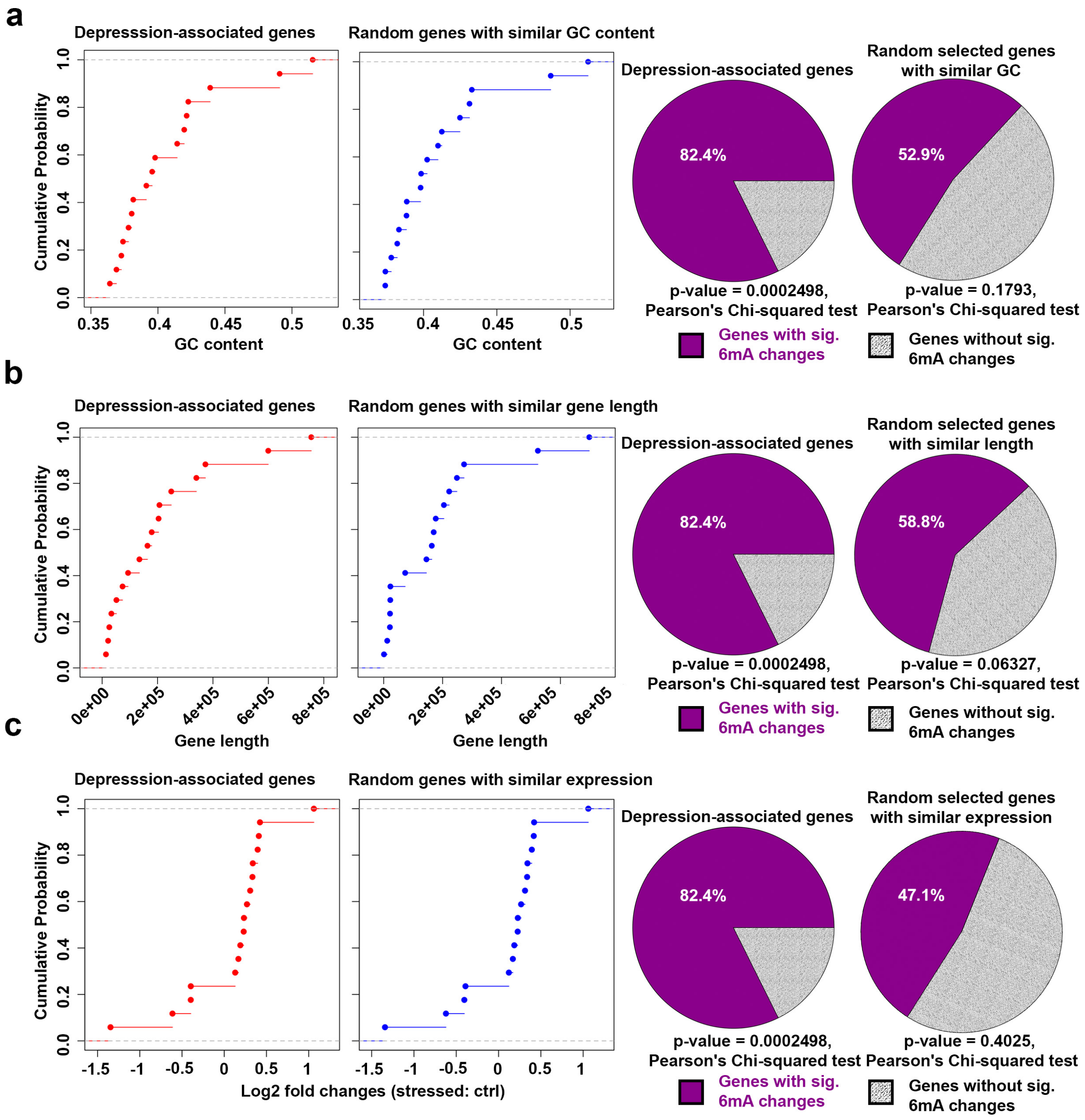

Supplementary Figure 11. 6mA correlated with depression-associated genes bearing 17 most significant SNPs. 17 depression-related genes with most significant SNPs, as well as total mouse coding genes were computed to separately retrieve their GC contents, gene length and expression dynamics upon stress. The empirical distribution of random genes from total mouse genes was set to match the characteristics with depression-related genes based on GC content (**a**), gene length (**b**) and expression dynamics (**c**). All three characteristics from two groups of depression-related genes and randomly selected genes were indicated in X-axis, and cumulative probability were indicated in Y-axis. None of the randomly selected genes showed significantly dynamic 6mA changes upon stress, whereas depression related genes displayed significant 6mA dynamics. Statistical significance was calculated by Pearson's Chi-squared tests, and p-values were indicated.

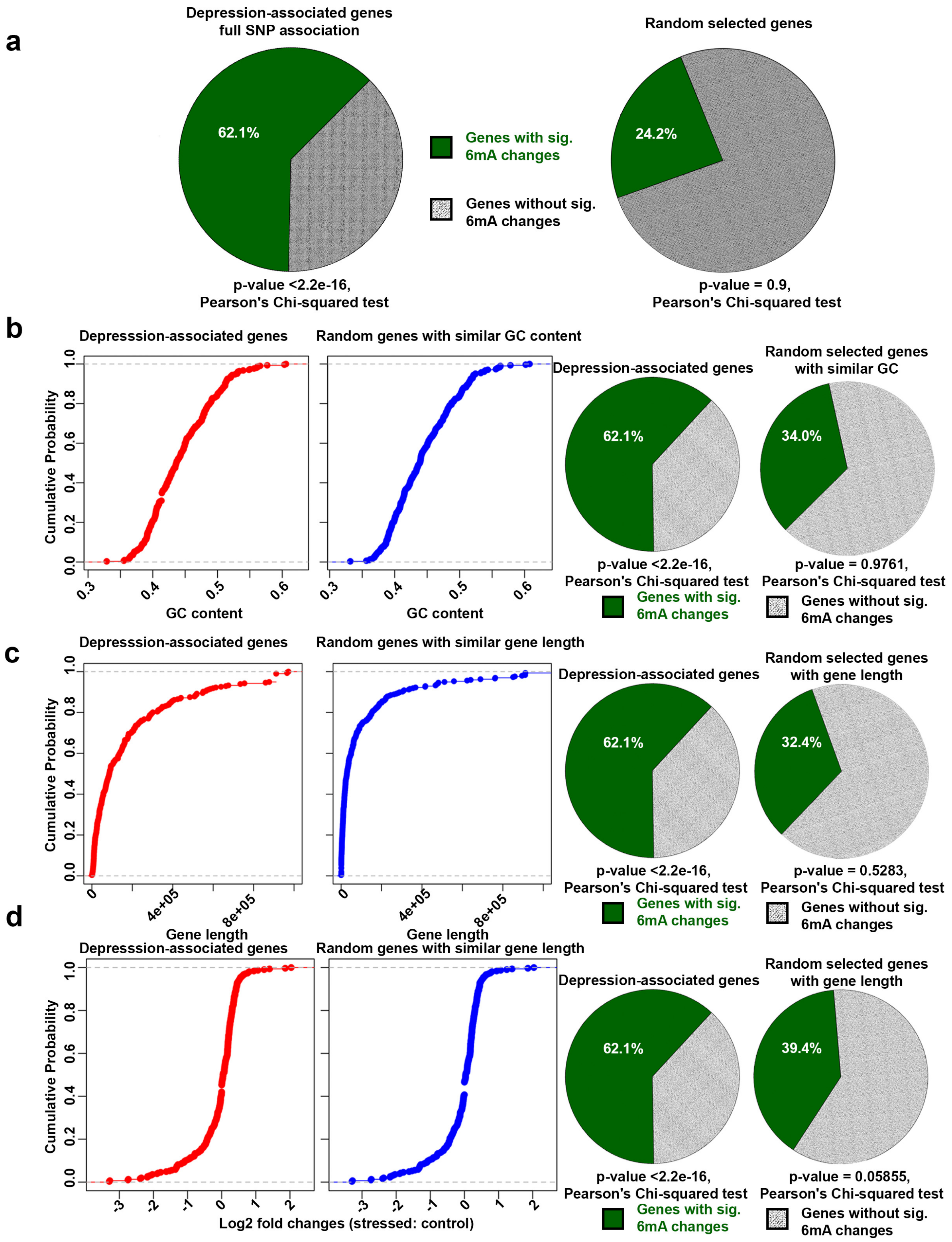

Supplementary Figure 12. 6mA is associated with depression-associated genes bearing top 10,000 intragenic significant SNPs. (**a**) Significant overlap between depression-associated genes containing the top 10,000 significant SNPs and dynamic 6mA marked genes upon stress

but not randomly selected genes were indicated. Statistical significance was calculated by Pearson's Chi-squared tests, and p values are indicated. (**b-d**) 309 depression-related genes bearing top 10,000 most significant depression associated SNPs, as well as total mouse coding genes were computed to separately retrieve their GC content, gene length and expression dynamics upon stress. The empirical distribution of random genes from total mouse genes was set to match characteristics with depression-related genes based on GC content (**b**), gene length (**c**) and expression dynamics (**d**). All three characteristics from two groups of depression-related genes and randomly selected genes were indicated in X-axis, and cumulative probability were indicated in Y-axis. None of the randomly selected genes showed significantly dynamic 6mA changes upon stress, whereas depression related genes displayed significant 6mA dynamics. Statistical significance was calculated by Pearson's Chi-squared tests, and p values are indicated.
