# Supplementary material for: DNA N6-methyladenine is dynamically regulated in the mouse brain following environmental stress
Source: Nat Commun. 2017 Oct 24;8:1122. doi: 10.1038/s41467-017-01195-y (PMC5654764; doi:10.1038/s41467-017-01195-y)
Supplement: Supplementary file 3 — Description of Additional Supplementary Files [file 41467_2017_1195_MOESM3_ESM.pdf]

File Name: Supplementary Data 1
Description: Significant loss-of-6mA regions upon stress.

File Name: Supplementary Data 2
Description: Significant gain-of-6mA regions upon stress.

File Name: Supplementary Data 3
Description: Stress-induced transposon expression.

File Name: Supplementary Data 4
Description: Stress-induced gene expression alterations determined by RNA-seq.

File Name: Supplementary Data 5
Description: Upregulated genes bearing significant loss-of-6mA are strongly associated with biological pathways.
